# When technology advances faster than competence: the risk of deskilling in obstetrics and gynecology

**DOI:** 10.61622/rbgo/2026rbgo37

**Published:** 2026-05-12

**Authors:** Agnaldo Lopes da Silva, Eduardo Cordioli, Sérgio Podgaec, Marcos Felipe Silva de Sá, Maria Celeste Osorio Wender

**Affiliations:** 1 Universidade Federal de Minas Gerais Belo Horizonte MG Brazil Universidade Federal de Minas Gerais, Belo Horizonte, MG, Brazil.; 2 Hospital e Maternidade Santa Joana São Paulo SP Brazil Hospital e Maternidade Santa Joana, São Paulo, SP, Brazil.; 3 Sociedade Beneficente Israelita Brasileira Albert Einstein São Paulo SP Brazil Sociedade Beneficente Israelita Brasileira Albert Einstein, São Paulo, SP, Brazil.; 4 Universidade de São Paulo Faculdade de Medicina São Paulo SP Brazil Faculdade de Medicina, Universidade de São Paulo, São Paulo, SP, Brazil.; 5 Universidade de São Paulo Faculdade de Medicina Ribeirão Preto SP Brazil Faculdade de Medicina, Universidade de São Paulo, Ribeirão Preto, SP, Brazil.; 6 Universidade Federal do Rio Grande do Sul Porto Alegre RS Brazil Universidade Federal do Rio Grande do Sul, Porto Alegre, RS, Brazil.

**Keywords:** Artificial Intelligence, Telemedicine, Digital protocols, Patient safety

## Introduction

A few years ago, at the beginning of the use of artificial intelligence (AI) applied to healthcare, the technology brought with it an uncomfortable contradiction. The more efficient the system, the less the doctor needed to think. And it is precisely at this point that the problem that this article proposes to discuss begins. AI is advancing at a speed that surpasses the capacity of healthcare institutions, medical schools, and the professionals themselves to adapt critically and safely. In Gynecology and Obstetrics (GO), this transformation is particularly visible: algorithms support the screening of pre-eclampsia risk, automated systems interpret cardiotocographies, machine learning models assist in the analysis of ultrasound images, and clinical decision support platforms are progressively incorporated into the care routine.^(1)^ For example, first-trimester screening algorithms combining maternal characteristics, mean arterial pressure, uterine artery Doppler and placental biomarkers are already used to estimate the individual risk of pre-eclampsia and guide prophylactic interventions. Similarly, machine-learning tools are being incorporated into cardiotocography interpretation and ultrasound image analysis, assisting clinicians in identifying fetal structures or estimating gestational age. These systems illustrate how algorithm-supported decision-making is progressively entering routine obstetric and gynecologic practice.

In this scenario, the gain in efficiency is undeniable. But there is a cost that rarely appears in technological validation articles: the progressive erosion of the doctor's independent clinical reasoning ability. This phenomenon has a name: deskilling. In a specialty where rapid decision-making, based on physical examination, detailed history, and contextualized interpretation of data, can define the lives of a mother and newborn, deskilling is not an academic debate. It is a real clinical problem. The objective of this study is to analyze the phenomenon of deskilling in Obstetrics and Gynecology in the context of artificial intelligence and digital health technologies, identifying its main drivers, potential implications for clinical practice, and strategies that may help preserve professional competence.

Modern Medicine has been reshaped by digitalization, standardized protocols, and increasing subspecialization. In this context, deskilling refers to the gradual erosion, or incomplete acquisition, of essential technical, cognitive, and interpersonal skills resulting from excessive reliance on automation and restricted clinical roles.^(2,3)^

Deskilling, in this context, does not result from individual inadequacy. It reflects a structural shift in practice, where automated outputs increasingly mediate interpretation and decision-making.^(2-4)^ Reduced exposure to complex cases, diminished reliance on physical examination, and habitual dependence on algorithmic recommendations may gradually weaken adaptive judgment, particularly in high-risk or unpredictable situations.^(4)^ A practical illustration of this shift is the trainee who postpones clinical judgment until the algorithmic risk score is available. Over time, waiting for the system's output may subtly replace the cognitive effort of integrating history, examination, and contextual data, not because of negligence, but because the system has repeatedly "worked well enough."

Technology can enhance medical work; however, it does not replace the need for clinical responsibility, situational awareness, and contextual interpretation in patient care.^(5,6)^ Too much reliance on AI could lead to automation bias, lower cognitive engagement, and a higher risk of making mistakes, especially among trainees and early-career clinicians who may never fully master basic skills.^(2,3)^ In this context, professional organizations have stressed that digital tools should enhance, not replace, clinical responsibility and ethical accountability in the care of patients.^(7)^

**Figure 1:**
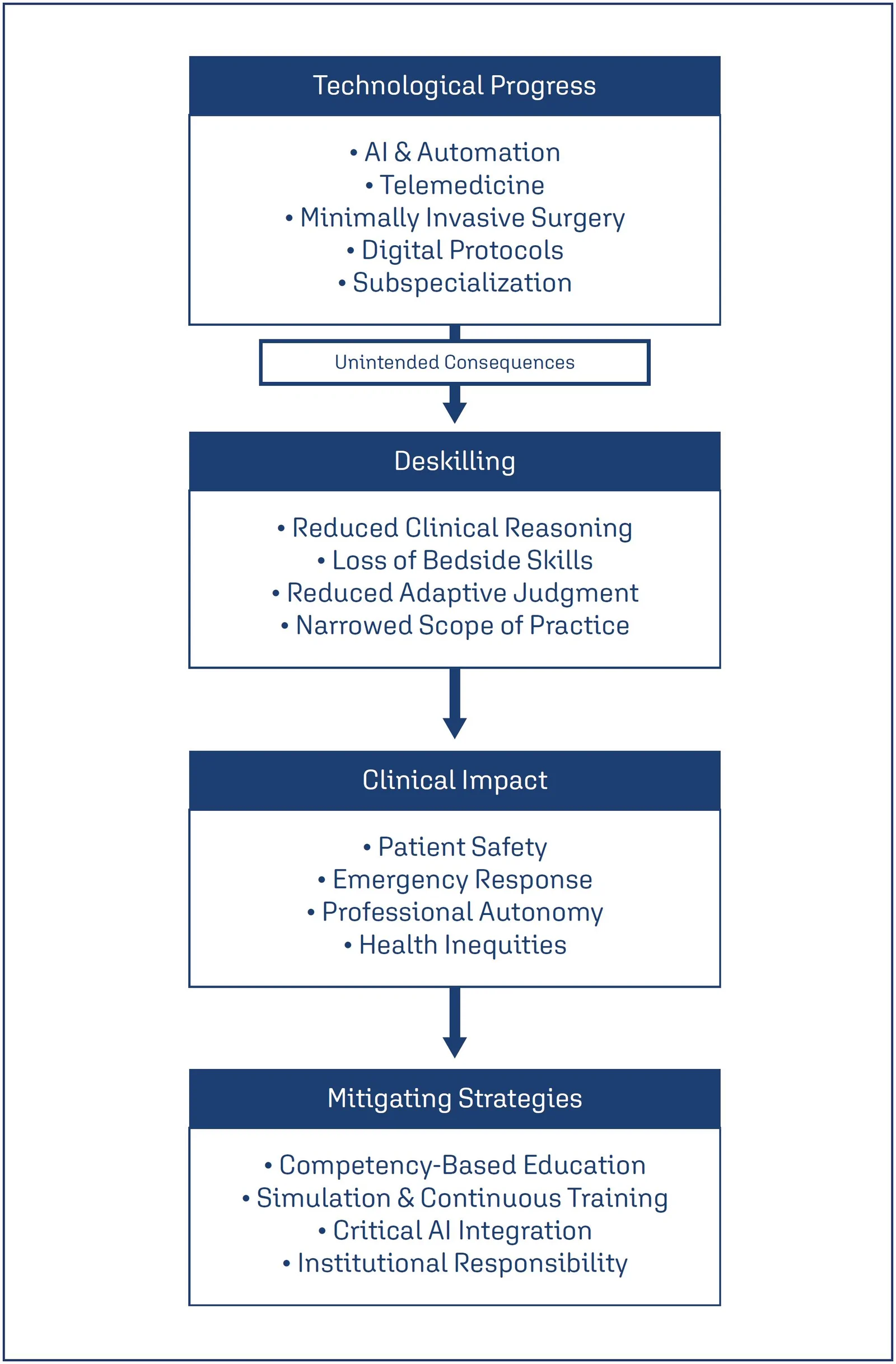
The paradox of technological progress: pathways to deskilling in Obstetrics and Gynecology

Note: Technological advances in Obstetrics and Gynecology, such as artificial intelligence, digital protocols, telemedicine, minimally invasive surgery, and increasing subspecialization, have improved efficiency and standardization of care. However, when not accompanied by deliberate educational and organizational strategies, these innovations may contribute to progressive deskilling, including reduced clinical reasoning, loss of bedside skills, and diminished adaptive judgment. The figure highlights the need for mitigating strategies, including competency-based education, simulation, and critical integration of digital technologies.

The challenge, therefore, is not technological progress itself, but how it is integrated into practice and training. Without deliberate educational and institutional strategies, efficiency gains may coexist with a gradual erosion of essential competencies, as illustrated in figure 1.

## Why is Obstetrics and Gynecology particularly susceptible to deskilling?

Obstetrics and Gynecology are particularly susceptible to deskilling due to a variety of structural, organizational, and technological factors. The way clinical practice is done has changed because of fast technological progress, the growth of minimally invasive techniques, more specialized fields, less time spent on hands-on training, and more pressure to be productive. As a result of these changes, people have had less and less exposure to complicated clinical situations and fewer chances to keep up their important surgical and clinical skills at all stages of their professional development.^(2,8,9)^

The fragmentation of care resulting from subspecialization has narrowed the scope of practice for numerous clinicians. While specialization has improved expertise in particular areas, it has also reduced procedural variety and continuity of care, particularly in gynecologic surgery and high-risk pregnancy. Because of this, both experienced specialists and doctors in training may have fewer chances to practice and improve the core skills that were once important to the specialty.^(8)^

Technological dependence is an increasingly significant factor contributing to deskilling. As the use of AI, automation, and protocol-based workflows continues to grow, clinical responsibility may shift from the active judgment of doctors to decisions made by the system. When technology supplants clinical reasoning rather than augmenting it, it can weaken the capacity for independent decision-making and adaptive judgment. This phenomenon affects both trainees and experienced specialists, especially as more and more tasks that used to be done by doctors are now being done by automated systems. ^(2,9)^ A practical illustration of this shift is the trainee who postpones clinical judgment until the algorithmic risk score is available. Over time, waiting for the system's output may subtly replace the cognitive effort of integrating history, examination, and contextual data, not because of negligence, but because the system has repeatedly "worked well enough."

## Drivers of deskilling in women's health

Several factors are contributing to the decline in women's health care quality. An overreliance on technology and algorithm-based decision support has gradually shifted diagnostic and management responsibilities away from active clinical reasoning. While artificial intelligence can improve efficiency, depending on it without careful thought may hinder independent decision-making, reduce mental engagement, and negatively affect communication and the doctor-patient relationship, especially for physicians who are still in the learning phase.^(2,4)^

Another amplifying factor is algorithmic opacity. Black-box systems that provide recommendations without making their reasoning transparent encourage uncritical deference, particularly when clinicians lack the technical literacy to interrogate the model's assumptions.^(10)^

Increasing subspecialization narrows scope of practice and reduces exposure to diverse clinical scenarios, limiting opportunities to maintain broad competence.^(11)^ Another important factor is less exposure to surgery. The number of cases is going down, minimally invasive techniques are becoming more popular, the demographics of patients are changing, and there are limits on training hours, all of which have made hands-on experience less common. In many training programs, the amount of practice is insufficient for trainees to develop and maintain proficiency in complex surgical skills, leading to lasting effects on their competence and confidence.^(8)^

Deskilling is also a result of excessive protocolization and a defensive approach to medical practice. While adhering to rules and checklists is crucial for ensuring safety and maintaining standardization, it can inadvertently hinder clinicians from exercising their own judgment. Over time, this can lead to a checklist-based approach to care, which makes it harder to think critically and make decisions in complex or unusual clinical situations.^(4,11)^

Changes in residency structure, including work-hour restrictions, administrative burden, and denser curricula, have reduced opportunities for supervised practice and mentorship, affecting both trainees and experienced clinicians.^(4,8)^ In the fields of obstetrics and gynecology, these factors contribute to a prevalent risk of deskilling that impacts both novice physicians and seasoned specialists.

## Why deskilling matters for patient care in women's health

The literature suggests that deskilling may have several implications for clinical practice in Obstetrics and Gynecology, particularly in settings that require rapid decision-making, procedural competence, and contextual interpretation of clinical information. Deskilling directly threatens patient safety by weakening independent clinical reasoning and adaptive judgment, particularly in complex or unexpected situations.^(2)^ This risk affects both trainees and experienced clinicians, especially when core responsibilities are progressively delegated to automated systems.^(2)^

Deskilling also has broader implications for the workplace. As clinicians rely more on standardized protocols and delegate tasks, their professional autonomy may decline, limiting the physician's ability to act as an independent decision-maker. This change can harm the relationship between the doctor and the patient, as communication, empathy, and trust—key components of Obstetric and Gynecologic care—are especially important in maternity care, where emotional support and trust are central to the physician–patient relationship.^(2)^ Deskilling has a clear clinical effect, especially in Obstetrics and Gynecology. In obstetric emergencies, delays in recognizing or managing complications can happen when skills and mental preparedness are not sufficiently maintained. In gynecologic surgery, fewer procedures and less exposure are linked to more complications and worse results. In environments where resources are scarce, it may be appropriate to delegate tasks to non-specialists for interventions that are high in volume but low in complexity. However, the insufficient presence of specialist-level expertise for more complex cases can threaten patient safety and adversely affect long-term outcomes.^(12)^

## Deskilling, health equity, and the circumstances in Latin America

Deskilling in low- and middle-income settings, including Brazil and other parts of Latin America, intersects directly with longstanding inequities in workforce distribution and access to continuing professional development. Clinical expertise remains concentrated in major urban centers, while rural and underserved regions face persistent shortages of specialists.^(13,14)^ In parallel, limited digital infrastructure, inadequate internet access, and disparities in digital literacy restrict both patient access to telehealth and clinicians’ opportunities for ongoing training.^(15,16)^ In Brazil, these disparities are particularly evident in the North and Northeast regions, where systemic issues hinder the effective implementation of telemedicine and digital education initiatives, limiting their accessibility to a broader audience.

In this context, deskilling does not merely reflect technological dependence, it risks amplifying structural inequalities. When highly trained specialists are scarce and complex decision-making is increasingly mediated by systems that are unevenly distributed or poorly supervised, the capacity to deliver context-sensitive, high-quality women's health care is compromised. Without coordinated policies addressing workforce allocation, digital inclusion, and sustained professional development, technological innovation alone will not translate into equitable health gains.^(13-15,17)^

## From awareness to action: maintaining clinical proficiency in Obstetrics and Gynecology

Preventing deskilling requires coordinated action from educators, professional societies, training institutions, and health systems. In the fields of obstetrics and gynecology, research indicates that deskilling is not a foregone conclusion nor an irreversible process, provided that deliberate strategies are employed to sustain clinical proficiency over the course of a professional career.

Competency-based medical education has become a key way to fill in gaps in training by shifting the focus from time-based exposure to clear clinical competence. In obstetrics and gynecology, competency-based models focus on mastering surgical skills, emergency management, and long-term care. This makes sure that the training outcomes match the needs of real-world clinical practice. Preliminary evidence suggests that these frameworks aid in the swift identification of at-risk learners and improve remediation efforts, which in turn influences patient safety and readiness for independent practice.^(18-21)^

Simulation-based education and ongoing professional development are important additions to clinical experience, especially in places where the number of procedures is going down. Simulation enables focused practice in rare, complex, or high-stakes scenarios and promotes the growth of both technical and interpersonal skills. Proficiency-based simulation curricula, which require students to show that they have mastered a skill instead of completing a set number of cases, have been shown to be better at transferring skills to clinical practice and helping students keep their skills over time in obstetrics and gynecology.^(22-24)^ Simulation is also very important at times when people are changing careers, as it helps keep skills sharp from residency to independent practice and later professional stages.^(23)^

It is important to be extra careful when adding technology to training and practice. AI and digital decision-support systems should help, not replace, clinical reasoning. Introducing decision-support tools before foundational clinical reasoning is consolidated risks producing operators of systems rather than autonomous physicians, much like learning to use a calculator before understanding arithmetic. To mitigate automation bias and maintain independent decision-making, it is essential to educate clinicians on how to critically assess and contextualize algorithmic outputs through both supervised and reflective engagement with technology.^(3)^

Without these kinds of protections, the chances of deskilling, not learning all the skills you need, or relying too much on automated recommendations go up a lot.

Maintaining comprehensive clinical practice is a significant challenge in a highly subspecialized setting. While subspecialization has enhanced care in specific fields, excessive fragmentation complicates the management of emergencies and intricate cases that require a diverse set of clinical skills. Training programs and health systems must ensure sufficient exposure to a range of clinical situations, particularly in obstetric emergencies and gynecologic surgery, where timely decision-making and skillful execution are critical elements that affect outcomes.^(20,21,23)^

Professional societies, educational institutions, and healthcare systems share the duty of maintaining standards of competence. Specialty societies establish benchmarks for proficiency, assist with certification processes, and provide ongoing education that adapts to advancements in the field. Training schools need to spend money on improving their teachers, building simulation infrastructure, and using competency-based tests. Health systems must then help keep skills up by giving people time to train, enough cases to work on, and rewards for continuing education.^(21,23,25,26)^

To prevent the decline of skills in obstetrics and gynecology, it is essential that we continue to collaborate consistently over time. Competency-based education, simulation, continuous training, crucial technology integration, and institutional accountability are not merely optional reforms; they are vital for upholding clinical excellence and ensuring safe, high-quality care for women. There is an urgent need for longitudinal studies evaluating whether sustained exposure to AI-assisted decision-making affects independent diagnostic accuracy and procedural competence over the course of a clinician's career.

## Final considerations

The rapid integration of technology in obstetrics and gynecology presents both a significant opportunity and a responsibility. Innovation has improved diagnostic accuracy, treatment options, and access to care, but it has also put the specialty at risk of losing skills over time. This has direct effects on patient safety, professional independence, and the long-term viability of clinical excellence. In daily life, this paradox does not present itself as a theoretical issue; rather, it gradually undermines clinical judgment, which typically becomes apparent only in emergencies. As digital tools, algorithms, and automation play a larger role in clinical workflows, the resulting decrease in time spent at the bedside and a reduced reliance on physical examinations and contextual reasoning may jeopardize the very foundations of clinical decision-making. This challenge is especially important in obstetrics and gynecology, a field that includes surgery, emergencies in pregnancy, and long-term women's health. In this vast and intricate domain, the capacity to integrate clinical findings, procedural knowledge, and situational judgment is crucial, particularly in unforeseen or high-risk situations where technology provides minimal assistance. In daily life, the impacts of this change tend to be minor and accumulate gradually, becoming evident only when immediate expert assistance is required but is not readily accessible. Not paying attention to the risk of deskilling is no longer a neutral position; it shows that medical education and professional regulation aren't working properly. It is important to keep core clinical skills because they are necessary for safe and ethical use of technology, not because they are against it. Algorithms and protocols cannot substitute for clinical judgment, procedural skill, or the capacity to make adaptable decisions. It is essential for educators, training institutions, health systems, and professional organizations to work together in prioritizing the upkeep of these competencies throughout an individual's professional career. In this situation, FEBRASGO plays a key role. The Federation stands as Brazil's premier scientific and professional organization for obstetrics and gynecology. It occupies a distinctive role in establishing standards for proficiency, endorsing evidence-based training models, and facilitating the crucial incorporation of technology into clinical practice. ^5,6^ This includes promoting education based on skills, encouraging simulation and ongoing professional development, and making sure that new ideas make clinical expertise stronger instead of replacing it. This responsibility is particularly crucial for maintaining the broadly trained obstetrician-gynecologist, who is equipped to manage emergencies and complex cases outside of highly specialized tertiary centers. Additionally, FEBRASGO is responsible for addressing the disparities in training environments and resources that exist throughout the country. By making sure that educational strategies, certification processes, and continuing education are in line with how things are done in real life today, the Federation can help reduce differences that may make people less skilled in areas that don't get enough help and make a big difference in protecting the quality of women's health care in Brazil. A constructive approach to this transition is to view artificial intelligence as a tool that augments clinical capabilities while preserving the physician's independent judgment as the central element of medical decision-making. The future of obstetrics and gynecology will depend a lot on finding the right balance between new ideas and old ones. Advancements in technology ought to enhance clinical judgment rather than diminish it. Ensuring that new technologies contribute to safer, fairer, and improved care for women, while preserving the essential skills that define the specialty, is achievable through a shared commitment and strong institutional leadership.

## Data Availability

The research data are described in the article presented.
